# Racial and Ethnic Differences in Satisfaction with Care Coordination Among VA and non-VA Medicare Beneficiaries

**DOI:** 10.1089/heq.2016.0012

**Published:** 2017-04-01

**Authors:** Sai K. Loganathan, Jennifer C. Hasche, Kevin T. Koenig, Samuel C. Haffer, Uchenna S. Uchendu

**Affiliations:** ^1^ NORC at the University of Chicago, Bethesda, Maryland.; ^2^ NORC at the University of Chicago, Chicago, Illinois.; ^3^ Data and Policy Analytics Group, Centers for Medicare & Medicaid Services, Baltimore, Maryland.; ^4^ United States Department of Veterans Affairs, Office of Health Equity, Washington DC.

**Keywords:** care coordination, health disparities, racial minority, veteran health

## Abstract

**Purpose:** Patients who have multiple sources of care are at risk for fragmented and uncoordinated care, which can lead to poorer outcomes. Veteran Medicare beneficiaries who use the Veterans Health Administration (VHA) system (VA users), particularly racial/ethnic minorities, often have complex medical conditions that may require care from multiple sources, leaving them especially vulnerable to the effects of fragmented care. We examined racial/ethnic differences in the level of satisfaction with care coordination among Medicare beneficiaries, comparing those who do and do not use the VHA healthcare system.

**Methods:** We conducted a retrospective, pooled, cross-sectional study of Medicare beneficiaries using the 2009–2011 Medicare Current Beneficiary Survey. The outcomes are self-reported satisfaction with care items related to three dimensions of care coordination: (1) integrated care, (2) care continuity, and (3) follow-up care. We present descriptive statistics and use generalized linear models to examine racial/ethnic differences across VA and non-VA users, after accounting for other demographic characteristics, health status, functional limitations, insurance coverage, and geographic variation.

**Results:** VA users are more likely to be very satisfied with receiving both integrated and follow-up care compared with non-VA users. Despite the existence of significant racial/ethnic disparities in the likelihood of being very satisfied with receiving well-coordinated care in the larger Medicare population, racial/ethnic minority VA users are just as likely as White non-Hispanics to be very satisfied with receiving well-coordinated care.

**Conclusions:** Future research should continue to study care coordination among VA users and reasons for preferring the VA over other healthcare systems, especially among racial/ethnic minority groups.

## Introduction

The Veterans Health Administration (VHA) is the largest integrated healthcare system in the United States, with close to 1700 sites of care, serving nearly 9 million veterans annually at a cost of around $55 billion in 2013.^1,2^ Although veterans may receive healthcare services exclusively at the VHA, many veterans receive healthcare services at both VHA and non-VHA facilities. Veterans, particularly Black non-Hispanic and Hispanic (minority) veterans, often have complex medical conditions that may require care from multiple sources, potentially leading to fragmented and uncoordinated care.^3,4^

Nearly all veterans 65 years and older (98%) qualify for Medicare,^5^ and prior analyses have shown that close to half of elderly veterans^6^ receive outpatient care from both VHA and Medicare financed facilities, with around 6% of elderly veterans receiving inpatient services at both VHA and non-VHA facilities.^7^ With passage of the Veterans Choice Act in 2014, which requires the VHA to offer non-VHA care for veterans who are unable to schedule an appointment within 30 days or reside >40 miles from the nearest VHA facility, veterans may be increasingly diversifying their care across multiple healthcare systems.^8^

Both providers^9^ and patients^10^ consider care continuity, care integration, and follow-up care after treatment as key aspects of high-quality patient care. Care continuity is generally conceptualized as “…the degree to which a series of discrete healthcare events is experienced as coherent and connected…,”^11^ whereas care integration is generally defined as the coordination of care in a single process across time, place, and discipline.^12^ Given that these dimensions of care coordination are associated with better care and health outcomes^13^ and reduced hospital admissions and re-admissions,^14^ there is concern that veterans who access multiple systems of care may experience fragmented and uncoordinated care, leading to worse outcomes.^15^ Analyses of veterans who use both Medicare and VHA inpatient or outpatient services found that dual (vs. single) system users experienced higher inpatient readmission rates after a hospitalization for acute stroke^16^ or heart failure,^17^ overused blood glucose test strips,^18^ increases in mortality risk,^19^ and experienced poorer quality of diabetes care.^20^ In contrast, other studies have found no difference between dual and VHA exclusive users across quality outcomes, such as cholesterol, blood pressure, hypertension, glycosylated hemoglobin and diabetes control,^21^ and number of ambulatory care sensitive hospitalizations.^22^

Research has shown considerable racial/ethnic disparities across multiple dimensions of health and healthcare, including outcomes such as blood pressure control and receipt of preventive care, among both VA (those who use the VHA system)^23^ and non-VA users (those who do not use the VHA system).^24^ Among Medicare beneficiaries overall, research has also shown racial/ethnic disparities in receipt of coordinated care. Black non-Hispanic and Hispanic minority patients are less likely to report healthcare from the same location and care continuity with the same provider, compared with White non-Hispanic beneficiaries,^25^ at the same time that minority patients, including minority VA users, are more likely to need treatment for complex conditions such as diabetes and end-stage renal disease.^3,4^ Although racial/ethnic disparities in care integration and care continuity among VA users have not been widely studied, one study found no difference in self-reported care continuity across race/ethnicity,^26^ although another study found that Black non-Hispanics were less likely to receive, related to care continuity, follow-up outpatient care for bipolar disorder.^27^

Given the aforementioned importance of measuring the extent to which VA users, in general, and racial/ethnic minority VA users, in particular, receive well-coordinated care, this research will address gaps in the literature by addressing the following questions:

(1) Are there differences between VA users' and non-VA users' self-reported satisfaction with receiving well-coordinated care?
(2) Are there racial/ethnic differences in satisfaction with care coordination among all Medicare beneficiaries?
(3) Among VA users, are there racial/ethnic differences in satisfaction with care coordination?

## Methods

### Study population

The Medicare Current Beneficiary Survey (MCBS) is an in-person, nationally representative, longitudinal rotating panel-design survey of Medicare beneficiaries that is sponsored by Centers for Medicare & Medicaid Services (CMS) and directed by the Office of Enterprise Data and Analytics (OEDA). The population for this study included full and part-year community-dwelling Medicare beneficiaries with Medicare entitlement at any point (“ever-enrolled”) during the calendar year, for each of the years 2009–2011. The study population, on average, accounts for 96% of all Medicare beneficiaries per year during 2009–2011. Medicare beneficiaries who were institutionalized for the entire calendar-year were excluded from the study as these beneficiaries do not receive the survey questions on satisfaction with care. Since the MCBS is based on a rotating panel design, pooling multiple years of data can result in respondents occurring one or more times in the analytic sample. We use balanced repeated replication (BRR) weights to account for overall selection probability of each sample beneficiary and include adjustments for the stratified sampling design based on age, sex, race/ethnicity, region, metropolitan area, survey nonresponse, coverage error, as well as the nonindependence of the beneficiary-years in the multi-year, pooled dataset. This results in nationally representative annual estimates of outcomes per beneficiary per year.

Medicare beneficiaries who qualify for VHA services may choose to receive healthcare services from the VHA, from Medicare-covered healthcare providers, or from both. Since the MCBS includes self-reported utilization information from all payment sources, we identify all Medicare beneficiaries with any self-reported cost associated with the VHA and identify them as VA users. More than 96% of VA users self-reported being veterans, whereas around 18% of the non-VA users self-reported being veterans. All sampled VA users had at least some non-VA-related healthcare expenditures during the study period. To test the sensitivity of the findings to an alternate definition of who qualifies as a VA user, we set the threshold for VHA costs at 33% of total healthcare expenditures (henceforth, referred to as “regular VA users”). We chose this threshold because, on average, VHA costs accounted for a third of the total annual healthcare expenditures for VA users. Regular VA users accounted for 42% of all VA users. Other studies have used a similar threshold of VHA utilization or costs as a proportion of total utilization or costs to define the study population.^22^ About 22% of community-dwelling Medicare beneficiaries were veterans, and more than one-fourth of them received at least some home care services through the VHA. In our analysis, we compared VA users with all community-dwelling Medicare beneficiaries who are non-VA users, which includes non-veterans as well as a small percentage of veterans. Due to sample size limitations, we were unable to limit the non-VA user group to only veterans. To test whether the observed differences in outcomes were attributable to veteran status as opposed to being attributable to receiving care at the VHA, we conducted a sensitivity analysis by limiting the sample to veteran Medicare beneficiaries and comparing outcomes between VA users and non-VA users within this subgroup.

### Main measures

The MCBS includes a rich set of survey questions that measure respondents' level of satisfaction with dimensions of care coordination. This study focuses on self-reported satisfaction with three specific dimensions of care coordination: satisfaction with receiving (1) integrated care, (2) care continuity, and (3) follow-up care after initial treatment. Self-reports of satisfaction with care continuity and ambulatory or outpatient care visits have been shown to have moderate concordance with administrative or claims-based measures.^28–30^ In this study, we used the following survey questions to analyze three dimensions of care coordination: (1) Satisfaction with integrated care is operationalized with the question: “Please tell me how satisfied you have been with getting all your healthcare needs taken care of at the same location.” (2) Satisfaction with a physician's engagement in ongoing healthcare management (care continuity) is operationalized with a survey question that asked respondents to rate the degree to which they agree or disagree with the following statement about services at their usual place of care: “Your doctor [Physician's name] has/the doctors at [Provider's name] have a complete understanding of the things that are wrong with you.” (3) Satisfaction with follow-up care after initial treatment is operationalized with the question: “Please tell me how satisfied you have been with the follow-up care you received after an initial treatment or operation.” These survey items have also been previously used to measure aspects of care coordination related to their use in this study.^29,31,32^

Response options for the satisfaction with care integration and follow-up care survey questions are “very satisfied,” “satisfied,” “dissatisfied,” “very dissatisfied,” or “not applicable.” Response options for the care continuity survey question are “strongly agree,” “agree,” “disagree,” “strongly disagree,” and “no experience.” This study focuses on the likelihood of respondents being very satisfied about or strongly agreeing to statements regarding the extent to which they receive well-coordinated care. We categorized “very satisfied” and “strongly agree” as positive outcomes, taking a conservative approach to defining satisfaction, in part since the care coordination outcome items may be vulnerable to the phenomenon of “acquiescence”^33^ (i.e., respondents may answer in a way that endorses the perceived assertion in the survey questions given the survey questions are phrased: “Please tell me *how satisfied* you have been…,” which may lead to respondents stating they are “satisfied”) and weak satisficing (respondents may answer with an agreeable option when a neutral option is not available).^34^

The distribution of responses across all three survey questions is such that more than 93% of respondents indicate being satisfied or very satisfied with the quality of their care, with more than 68% of the respondents stating that they are satisfied. The distribution of responses supports other research that has found that patient satisfaction with healthcare is generally highly skewed toward high rates of satisfaction.^35^ Thus, we dichotomize the outcome variables with “very satisfied” or “strongly agree” coded as a positive outcome and all other responses otherwise, an approach adopted by other researchers.^36,37^ We exclude respondents who indicate having “no experience,” who state that the survey questions are “not applicable” to them, or for whom data are missing.

### Covariates

We define race/ethnicity as White non-Hispanic, Black non-Hispanic, Hispanic, and Other non-Hispanic. Due to the sample size of the MCBS, separate categories for American Indian or Alaska Native, Asian, and Native Hawaiian or Other Pacific Islander are not constructed. Gender is coded as a dichotomous variable, with female as the reference group. To control for differences in health status and functioning, we used information on self-reported health status, including the presence of specific chronic and acute health conditions (heart disease, cancer, hypertension, diabetes, mental illnesses, and stroke), and the number of limitations in activities of daily living (ADLs) and instrumental activities of daily living (IADLs). ADLs are often referred to as “self-care” limitations where a beneficiary reported that, because of a health or physical problem, he/she finds it difficult or is unable to bathe, shower, dress, eat, get in or out of bed or chairs, or use the toilet. IADLs are often referred to as “independent living” disabilities where a beneficiary reported that, because of a health or physical problem, he/she finds it difficult or is unable to shop for personal items, prepare own meals, manage money, use the telephone, or do housework. To account for variations in insurance coverage, we include covariates to identify any Medicare Advantage (MA), Medicaid, or private (Medigap, self-purchase, or employer-provided) coverage during the calendar-year. We account for geographic variation with both metro/non-metro and Census region indicators. To address missing data for covariates, a missing indicator category was created for each covariate and included in the outcome models to preserve the entire sample.

### Analysis

Throughout, our unit of analysis is a beneficiary-year, and our outcome is representative of satisfaction with care coordination per beneficiary per year. All estimates are weighted by using cross-sectional BRR weights (Fay's method)^38^ to represent the population of all “ever-enrolled” Medicare beneficiaries. We use sub-population (domain) analysis to analyze differences within and across subgroups. Using sub-population analysis within survey functions, we used survey-weighted, logistic regression models to estimate the odds of a respondent indicating “very satisfied”/“strongly agree” versus all other responses after excluding “not applicable” and “no experience” responses. To assess differences in outcomes among VA users in contrast to non-VA users, we included an indicator variable for VA user status. To assess racial/ethnic differences among all community-dwelling Medicare beneficiaries, we included a categorical variable for race/ethnicity, with White non-Hispanic as the base category. To assess racial/ethnic differences among VA users in contrast to the differences among non-VA users, we included interaction terms of the two covariates in the multivariate models. In addition, the models included covariates for other demographic factors, socioeconomic status, health status and functioning, insurance coverage, and geographic variation. We present the results of the multivariate logistic regressions as predicted probabilities (average marginal effect). We use a similar approach to model racial/ethnic differences in outcomes among the subset of regular VA users. All data manipulation and analyses were performed by using SAS 9.4 and Stata 13 software.

## Results

At least three-quarters of the study population indicated their level of satisfaction with integrated care, care continuity, and follow-up care after initial treatment (77.8%, 89.1%, and 82.3%, respectively; Table 1). The remaining respondents indicated having no experience with these quality dimensions; the study populations for the three outcomes do not differ significantly from one another, or from the larger ever-enrolled, community-dwelling Medicare population.

**Table 1.** **Percent of Study Population with Eligible Responses to Dimensions of Care Coordination Outcome Measures Among Community-Dwelling Medicare Beneficiaries, by Veterans Affairs User Status (Medicare Current Beneficiary Survey 2009–2011)**

Population	VA user beneficiary-years weighted (unweighted)	Non-VA user beneficiary-years weighted (unweighted)	Total beneficiary-years weighted (unweighted)
All survey respondents	8,537,136 (1,801)	137,069,052 (30,700)	145,606,188 (32,501)
Community-dwelling Medicare beneficiaries	8,499,039 (1,786)	131,383,033 (28,612)	139,882,072 (30,398)
Percent of survey respondents with eligible responses to the question on satisfaction with integrated care	84.2% (83.0%)	77.4% (77.4%)	77.8% (77.7%)
Percent of survey respondents with eligible responses to the question on satisfaction with care continuity	92.0% (91.7%)	88.9% (88.3%)	89.1% (88.5%)
Percent of survey respondents with eligible responses to the question on satisfaction with follow-up care	86.1% (86.0%)	82.0% (81.8%)	82.3% (82.1%)

VA users accounted for 6.1% of the study population. About 43% of VA users are regular VA users, and on average, the cost of these services amounted to 63% of a beneficiary's total annual healthcare expenditures. Table 2 presents demographic characteristics, socioeconomic status, insurance coverage, geographic variation, health status, and functional limitations across community-dwelling beneficiaries included in each outcome of interest.

**Table 2.** **Demographics of Community-Dwelling Medicare Beneficiaries: Veterans Affairs Users and Non-Veterans Affairs Users (Medicare Current Beneficiary Survey 2009–2011)**

	Community-dwelling Medicare beneficiaries	
Demographics	VA users % (95% CI)	Non-VA users % (95% CI)
Count of beneficiary-years (weighted)	8,499,039	131,383,033
Count of beneficiary-years (unweighted)	1,786	28,612
White non-Hispanic	79.4 (76.6–82.3)	76.7 (75.7–77.7)
Black non-Hispanic	11.1 (9–13.1)	9.5 (9.1–9.9)
Hispanic	5.8 (4.5–7.1)	9 (8.4–8.6)
Other	3.7 (2–5.5)	4.8 (4.2–5.5)
Gender		
Female	6.1 (4.6–7.6)	57.6 (57–58.2)
Male	93.9 (92.4–95.4)	42.4 (41.8–43)
Age category, years		
<65	20.8 (18.2–23.3)	17.9 (17.4–18.4)
65–74	36.1 (33.0–39.1)	43.5 (42.9–44.2)
75–84	28.8 (26.2–31.5)	27.1 (26.6–27.7)
>85	14.3 (12.5–16.2)	11.4 (11.0–11.9)
Income		
<$25,000	42.8 (40.4–45.3)	48.6 (47.7–49.4)
≥$25,000	57.2 (54.7–59.6)	51.4 (50.6–52.3)
Education		
No high school diploma	17.2 (14.8–19.7)	23.5 (22.6–24.4)
High school diploma and higher	82.8 (80.3–85.2)	76.5 (75.6–77.4)
Marital status		
Other	36.8 (34.1–39.4)	48.3 (47.5–49.1)
Married	63.2 (60.6–65.9)	51.7 (50.9–52.5)
Institutionalization		
No institutionalization	98.9 (98.5–99.3)	98.7 (98.6–98.8)
Part-year institutionalization	1.1 (0.7–1.5)	1.3 (1.2–1.4)
Metro region		
Non-metro	28.0 (24.5–31.6)	23.2 (22.8–23.6)
Metro	72.0 (68.4–75.5)	76.8 (76.4–77.2)
Insurance		
Medicaid	6.4 (4.8–8.0)	19.7 (19.0–20.4)
Medicare advantage	20.8 (18.4–23.1)	32.5 (31.6–33.4)
Private insurance	45.9 (42.7–49.1)	54.2 (53.3–55.0)
Functional limitations (ADLs/IADLs)		
No ADLs/IADLs	60.1 (57.1–63.0)	61.9 (61.0–62.7)
One to two ADLs/IADLs	24.7 (22.5–26.9)	23.7 (23.1–24.4)
Three or more ADLs/IADLs	15.2 (13.3–17.1)	14.4 (13.8–15.1)
Self-report of health status		
Excellent	13.5 (11.7–15.3)	15.6 (14.8–16.3)
Very good	24.6 (22.3–26.8)	28.5 (27.8–29.1)
Good	32.9 (30.3–35.4)	29.9 (29.2–30.7)
Fair	19.9 (17.3–22.5)	17.4 (16.9–17.9)
Poor	8.2 (6.5–9.9)	7.4 (7.0–7.8)
Unavailable	1 (0.6–1.4)	1.2 (1.1–1.3)
Disease conditions		
Mental condition	32.3 (29.0–35.6)	29.1 (28.2–30.0)
Diabetes	33.7 (31.1–36.3)	24.8 (24.0–25.6)
Hypertension	74.2 (71.6–76.7)	66.8 (65.8–67.8)
Heart disease	48.6 (45.2–52.0)	38.7 (37.8–39.6)
Stroke	14.0 (11.5–16.6)	10.5 (10.0–11.1)
Cancer	22.0 (19.3–24.8)	18.2 (17.5–18.9)
Census region		
North East	14.1 (11.7–16.4)	18.9 (18.3–19.5)
Mid-west	25.4 (22.3–28.5)	22.2 (21.6–22.9)
South	40.0 (36.6–43.5)	37.1 (36.3–37.9)
West	18.1 (15.4–20.9)	20.1 (19.4–20.8)
Puerto Rico	1.8 (1.5–2.1)	1.5 (1.4–1.7)
Other/unavailable	0.6 (0.0–1.1)	0.1 (0.1–0.2)
Year		
2009	34.1 (32.2–36.0)	32.3 (32.1–32.5)
2010	31.1 (29.4–32.8)	33.4 (33.2–33.5)
2011	34.8 (32.7–36.8)	34.4 (34.2–34.6)

Due to the sample size of the MCBS, separate categories for American Indian or Alaska Native, Asian, and Native Hawaiian or Other Pacific Islander are not constructed.

ADLs, activities of daily living; IADLs, instrumental activities of daily living.

Compared with non-VA users, VA users were more likely to be men; more likely to have an annual income at or above $25,000; more like to have graduated from high school; more likely to be married; less likely to live in a metropolitan area; less likely to be enrolled in Medicaid or MA; and more likely to report having diabetes, hypertension, heart disease, stroke, or cancer. To assess the racial/ethnic differences attributable to the differential impact of the healthcare system on the outcomes, factors associated with health status, clinical appropriateness need to be accounted for. To account for such factors, the multivariate models in this study include the covariates listed in Table 2.

**FIG. 1.**
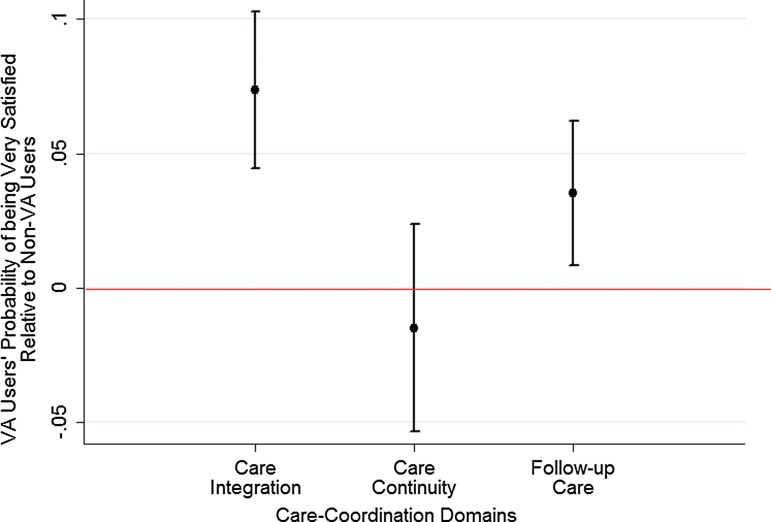
Satisfaction with care coordination: Veterans Affairs users compared with non-Veterans Affairs users. (Medicare Current Beneficiary Survey 2009–2011).

**FIG. 2.**
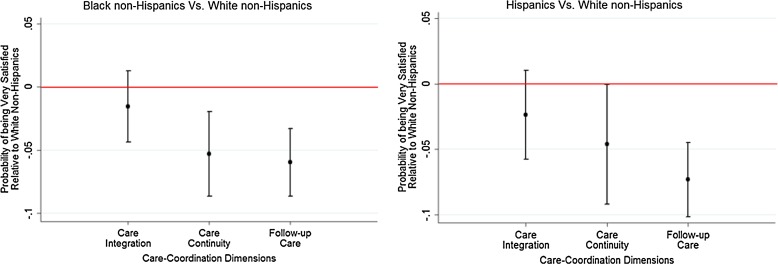
Racial/ethnic differences in satisfaction with care coordination among all Medicare beneficiaries. (Medicare Current Beneficiary Survey 2009–2011).

**FIG. 3.**
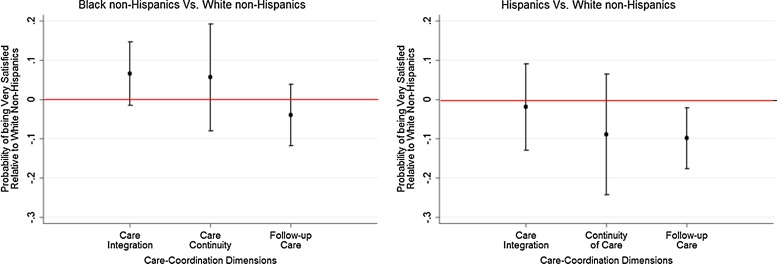
Racial/ethnic differences in satisfaction with care coordination among Veterans Affairs users. (Medicare Current Beneficiary Survey 2009–2011).

Regression estimates are presented as odds ratios (Table 3). To facilitate ease of interpretation of results in the presence of an interaction term, we present predicted probabilities as average marginal effects (AME) in Figures 1–3. As shown in Figure 1, a higher proportion of VA users are very satisfied with receiving integrated care ([AME]=7.4%; confidence interval [95% CI], 4.5%–10.3%) and follow-up care after initial treatment (AME=3.5%; 95% CI, 0.8%–6.2%) compared with non-VA users. No statistically significant differences exist between VA users and non-VA users in their likelihood of being very satisfied with care continuity (AME=−1.5%; 95% CI, −5.3% to 2.4%).

**Table 3.** **Multivariate Logistic Regression Analysis: Odds Ratios for Dimensions of Care Coordination (Medicare Current Beneficiary Survey 2009–2011)**

	Care integration		Care continuity		Follow-up care	
Variable	Unadjusted	Adjusted	Unadjusted	Adjusted	Unadjusted	Adjusted
VA user (non-VA user is the base category)	1.344^***^	1.370^***^	0.877	0.906	1.106	1.131
Black non-Hispanic (White non-Hispanic is the base category)	0.851^**^	0.952	0.740^***^	0.827^***^	0.609^***^	0.757^***^
Hispanic (White non-Hispanic is the base category)	0.759^***^	0.822^**^	0.779^***^	0.840^*^	0.611^***^	0.688^***^
Other (White non-Hispanic is the base category)	0.875	0.915	0.844	0.911	0.647^***^	0.724^***^
Black non-Hispanic VA user (White non-Hispanic non-VA user is the base category)	1.461^*^	1.388^*^	1.648^*^	1.474	1.269	1.130
Hispanic VA user (White non-Hispanic non-VA user is the base category)	1.137	1.198	0.686	0.596	1.160	0.982
Other VA user (White non-Hispanic non-VA user is the base category)	0.646	0.781	0.706	0.707	1.701	1.860^*^
Age		1.187^***^		1.035		1.244^***^
Male (vs. female)		1.127^*^		1.008		1.020
Married vs. (single/divorced/separated)		1.195^**^		0.974		0.909
Income >$25,000 (vs. income ≤$25,000)		1.062		0.952		1.040
High school or more (vs. no high school completion)		1.022		1.021		1.012
Any institutionalization		1.154^***^		1.226^***^		1.322^***^
Medicaid coverage		1.132^***^		1.205^***^		1.380^***^
Medicare advantage coverage		0.759		1.182		0.559^*^
Private/ESI coverage		1.073		1.002		1.043
One ADL/IADL limitation (vs. No ADL/IADL limitations)		1.149^***^		0.917^*^		0.954
Two or more ADL/IADL limitations (vs. No ADL/IADL limitations)		1.024		1.053		1.141^***^
Heart disease		0.816^***^		0.917^*^		0.868^***^
Hypertension		0.707^***^		0.985		0.802^***^
Diabetes		0.859^***^		0.988		0.954
Stroke		1.064		1.039		0.989
Any cancer		1.058		1.069		1.057
Any mental condition		0.997		0.970		1.018
“Very good” self-reported health (vs. “excellent”)		0.993		0.984		1.143^***^
“Good” self-reported health (vs. “excellent”)		0.822^***^		0.859^***^		0.807^***^
“Fair” self-reported health (vs. “excellent”)		1.292^***^		1.356^***^		1.321^***^
“Poor” self-reported health (vs. “excellent”)		1.313^***^		1.210^***^		1.131^*^
Not available/don't know/refused self-reported health (vs. “excellent”)		0.914		1.002		0.937
Living status—metro		1.204^***^		1.113		1.209^***^
Living status—Mid-west (vs. North East)		0.592^***^		1.240		1.262^**^
Living status—South (vs. North East)		1.592		1.370		1.200
Living status—West (vs. North East)		1.021		1.035		1.076^**^
Living status—Puerto Rico (vs. North East)		1.090^*^		1.141^***^		1.099^**^
Living status—Unavailable (vs. North East)						
Constant	0.320^***^	0.291^***^	0.689^***^	0.504^***^	0.447^***^	0.367^***^
N (sub-population)	23,631	23,631	26,900	26,900	24,952	24,952

We use balanced repeated replication weights to account for overall selection probability of each sample person and include adjustments for the stratified sampling design based on age, sex, race/ethnicity, region, metropolitan area, survey nonresponse, coverage error, as well as the nonindependence of the person-years in the multi-year, pooled dataset.

^*^ *p*<0.10, ^**^p<0.05, ^***^p<0.01.

To test the sensitivity of the findings to an alternate definition of who qualifies as a VA user, we limited the analysis to non-VA users and “regular” VA users. Results of the sensitivity test revealed that, similar to all VA users, regular VA users are also more likely to be very satisfied with receiving integrated care (AME=14.2%; 95% CI, 9.3%–19.1%) and follow-up care after initial treatment (AME=7.3%; 95% CI, 2.6%–12%) compared with all other beneficiaries. No statistically significant differences exist between regular VA users and non-VA users in their likelihood of being very satisfied with care continuity (AME=0.8%; 95% CI, −5.1% to 6.6%).

To test whether the difference in satisfaction with receiving well-coordinated care was attributable to the VA-users' veteran status as opposed to being attributable to receiving care at the VHA, we limited the study population to veteran, community-dwelling Medicare beneficiaries and compared differences in outcomes between VA users and non-VA users. Among veteran Medicare beneficiaries, VA users are more likely to be very satisfied with receiving integrated care (AME=3.7%; 95% CI, 0.1%–7.2%) compared with non-VA users. There were no statistically significant differences in the likelihood of being very satisfied with care coordination (AME=−3.1%; 95% CI, −7.5% to 1.3%) and follow-up care (AME=0%; 95% CI, −4.0% to 2.8%) between veteran VA users and veteran non-VA users.

After controlling for demographic, clinical, functional, and geographic differences, we find significant racial/ethnic differences among all beneficiaries in their likelihood of being very satisfied with care continuity and follow-up care. Black non-Hispanic beneficiaries are less likely to be very satisfied with care continuity (Fig. 2, AME=−5.3%; 95% CI, −8.7% to −1.95%) and follow-up care (AME=−6%; 95% CI, −8.6% to −3.3%) compared with White non-Hispanic beneficiaries. Hispanic beneficiaries are also less likely to be very satisfied with their care continuity (AME=−4.6%; 95% CI, −9.2% to −0.3%) and follow-up care (AME=−7.3%; 95% CI, −10.1% to −4.5%) compared with White non-Hispanic beneficiaries.

**FIG. 4.**
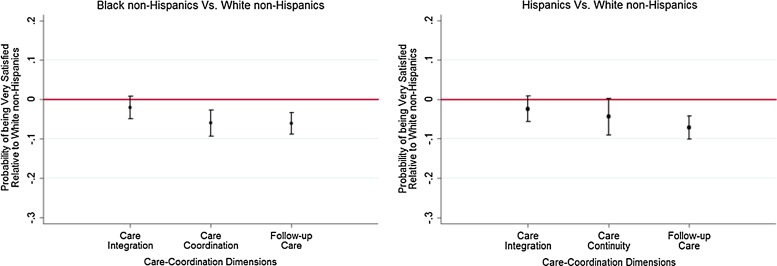
Racial/ethnic differences in satisfaction with care coordination among non-Veterans Affairs users. (Medicare Current Beneficiary Survey 2009–2011).

Small sample sizes limit our ability to detect racial/ethnic differences among VA users, with a high degree of certainty. We did not find a statistically significant difference between Black non-Hispanic VA users and White non-Hispanic VA users in being very satisfied with receiving integrated care (AME=6.6%; 95% CI, −1.5% to 14.7%) and care continuity (AME=5.7%; 95% CI, −7.9% to 19.3%; Fig. 3). In contrast, Black non-Hispanic non-VA users are less likely to be very satisfied with care continuity (AME=−6.0%; 95% CI, −9.3% to −2.7%) and follow-up care (AME=−6.1%; 95% CI, −8.9% to −3.3%). Among both VA users and non-VA users, Hispanics are less likely to be very satisfied with follow-up care after initial treatment compared with White non-Hispanics. There were no statistically significant differences in the likelihood of being very satisfied with receiving integrated care and care continuity between Hispanic non-VA users and White non-Hispanic non-VA users (Fig. 4). Although regular VA users, in general, tend to be more satisfied with care integration and follow-up care after initial treatment, racial/ethnic differences among regular VA users are similar to those of other VA users. Limiting the study population to veteran Medicare beneficiaries and assessing racial/ethnic differences between VA users and non-VA users resulted in similar findings. However, among veterans, Hispanic VA users were much less likely to be very satisfied with follow-up care compared with White non-Hispanics.

## Discussion

Our results show that despite concerns that VA users may receive fragmented care due to accessing care from multiple providers in more than one system, they report higher satisfaction with dimensions of care coordination once engaged with the VHA system. Specifically, VA users are more likely to be very satisfied with receiving integrated care and follow-up care, compared with non-VA users. Results of the sensitivity analysis showed that veteran VA users were more likely to be very satisfied with receiving integrated care than non-VA users, which further supports our hyposthesis that there are differences in satisfaction with care between those who do and those do not receive care from the VHA. Among Medicare beneficiaries who do not use the VHA, minority beneficiaries were less likely to be satisfied with receiving integrated care, care coordination, and follow-up care compared with White non-Hispanics. In contrast, both Black non-Hispanic and Hispanic VA users are as likely as White non-Hispanic VA users to be satisfied with receiving integrated care and care coordination. Our findings are consistent with prior research. VA users have been shown to be, in general, very satisfied with their care.^39^ They have also reported being more satisfied with the quality of their care than a comparison of a commercial population,^40,41^ which matches chart-based assessments and claims analyses of VA versus non-VA quality of care.^42,43^

Prior research has identified racial/ethnic disparities among VA users, with many of these studies demonstrating disparities in the treatment of invasive procedures, pain management, preventive care, and medication adherence.^23^ Few studies have assessed racial/ethnic disparities in dimensions of care coordination, or satisfaction generally, among VA users, but those have found mixed results. Some studies have found that Black non-Hispanic VA users report lower levels of patient satisfaction^44,45^ and are less likely to receive follow-up outpatient care,^27^ although studies have found little or no disparity in satisfaction of care or care coordination between White and Black non-Hispanic VA users.^46,47^ Our finding that there are smaller disparities in satisfaction with dimensions of care coordination among VA users adds to the literature. An important consideration is that Black non-Hispanics are more likely to use the VHA for their sole source of care,^48,49^ and furthermore, that Black non-Hispanic veterans, as well as Hispanic veterans, report that they preferred the VA to other healthcare systems.^50^

For more than a decade, federal health programs have focused on providing more integrated care and improving care coordination as a means to improve health outcomes, ensure patient safety, reduce healthcare costs, and strengthen health equity.^51^ However, recent data released by CMS continues to document significant racial and ethnic disparities in the care coordination experiences of beneficiaries enrolled in the MA and Medicare fee-for-service (FFS) programs.^52,53^ As care provided through alternate payment models continues to expand, there is an increasing need to identify, document, and disseminate evidence-based interventions with proven effectiveness at improving quality of care and reducing health disparities. Medicare FFS and MA providers reimbursed through alternate payment models may be able to improve the quality of care and reduce health disparities by adopting some of these care coordination approaches implemented by VHA.

There are limitations to this study. First, the MCBS relies on self-reported healthcare events and imputation to determine VHA utilization and costs. Since costs attributable to the VHA were used to identify VA users, the study population may have been under-represented.^54^ Second, the survey items comprising the care coordination dimensions may suffer from the phenomenon of acquiescence and/or weak satisficing; however, we used a conservative approach for measuring satisfaction to counteract these phenomenon. Third, it is not clear whether the self-reported satisfaction outcome measures are reflective of quality of care provided by one or both systems of care (Medicare-covered providers and the VHA). Finally, due to sample size limitations, this study was not able to assess how VA users who used the VHA predominantly for their care differed from other VA users.

## Conclusion

Our findings indicate that VA users, compared with non-VA users, report higher satisfaction with multiple dimensions of care coordination; and among VA users, minority beneficiaries are as satisfied as White beneficiaries with care integration and care continuity. Our study is significant in that it documents health equity in patient-reported measures of satisfaction with care coordination within a major federal health program. Given recent interest in policies aimed at increasing options for accessing care, such as the Veterans Choice Act, it will be important to continue to assess care coordination and potential fragmentation of care among VA users.

Future research should continue to study care coordination among VA users and reasons for preferring the VA over other healthcare systems, especially among racial/ethnic minority groups. Research should aim at identifying what care processes and interventions that have been implemented within the VA may be contributing to these results, and at determining the applicability and scalability to other healthcare settings, systems, and programs. In addition, studies that link satisfaction with health outcomes will help to further shape policies that are directed toward reducing disparities and improving health equity for all.

## Abbreviations Used

ADLs: activities of daily living
AME: average marginal effects
BRR: balanced repeated replication
CMS: Centers for Medicare & Medicaid Services
IADLs: instrumental activities of daily living
MA: Medicare Advantage
MCBS: Medicare Current Beneficiary Survey
OEDA: Office of Enterprise Data and Analytics
VA: Veterans Affairs
VHA: Veterans Health Administration
